# Intrahepatic *TLR3* and *IFNL3* Expressions Are Associated with Stages of Fibrosis in Chronic Hepatitis C

**DOI:** 10.3390/v13061103

**Published:** 2021-06-09

**Authors:** Keyla Santos Guedes de Sá, Ednelza da Silva Graça Amoras, Simone Regina Souza da Silva Conde, Maria Alice Freitas Queiroz, Izaura Maria Vieira Cayres-Vallinoto, Ricardo Ishak, Antonio Carlos Rosário Vallinoto

**Affiliations:** 1 Laboratory of Virology, Institute of Biological Sciences, Federal University of Pará, Belém 66.075-110, PA, Brazil; keyla-sa@hotmail.com (K.S.G.d.S.); ednelza@ufpa.br (E.d.S.G.A.); alicefarma@hotmail.com (M.A.F.Q.); ivallinoto@ufpa.br (I.M.V.C.-V.); rishak@ufpa.br (R.I.); 2 Graduate Program in Biology of Infectious and Parasitic Agents—PPG-BAIP, Institute of Biological Sciences, Federal University of Pará, Belém 66.075-110, PA, Brazil; 3 João de Barros Barreto University Hospital, Federal University of Pará, Belém 66073-000, PA, Brazil; sconde@ufpa.br; 4 School of Medicine, Institute of Health Sciences, Federal University of Pará, Umarizal, Belém 66.075-110, PA, Brazil

**Keywords:** hepatitis C virus, TLR3, IFN-λ3, gene, expression, inflammation, fibrosis

## Abstract

An inefficient immune response against the hepatitis C virus (HCV), combined with viral evasion mechanisms, is responsible for the chronicity of infection. The need to evaluate the innate mechanisms of the immune response, such as TLR3 and IFN-λ3, and their relationship with the virus–host interaction is important for understanding the pathogenesis of chronic hepatitis C. The present study aimed to investigate the gene expressions of *TRL3* and *IFNL3* in liver tissue, seeking to evaluate whether these could be potential biomarkers of HCV infection. A total of 23 liver biopsy samples were collected from patients with chronic HCV, and 8 biopsies were collected from healthy control patients. RNA extraction, reverse transcription and qPCR were performed to quantify the relative gene expressions of *TLR3* and *IFNL3*. Data on the viral load; AST, ALT, GGT and AFP levels; and the viral genotype were collected from the patients′ medical records. The intrahepatic expression of *TLR3* (*p* = 0.0326) was higher in chronic HCV carriers than in the control group, and the expression of *IFNL3* (*p* = 0.0037) was lower in chronic HCV carriers than in the healthy control group. The expression levels of *TLR3* (*p* = 0.0030) and *IFNL3* (*p* = 0.0036) were higher in the early stages of fibrosis and of necroinflammatory activity in the liver; in contrast, *TLR3* and *IFNL3* expressions were lower in the more advanced stages of fibrosis and inflammation. There was no correlation between the gene expression and the serum viral load. Regarding the initial METAVIR scale scores, liver transaminase levels were lower in patients with advanced fibrosis when correlated with *TLR3* and *IFNL3* gene expressions. The results suggest that in the early stages of the development of hepatic fibrosis, TLR3 and IFN-λ3 play important roles in the antiviral response and in the modulation of the tolerogenic liver environment because there is a decrease in the intrahepatic expressions of *TLR3* and *IFNL3* in the advanced stages of fibrosis, probably due to viral evasion mechanisms.

## 1. Introduction

The estimated global prevalence of hepatitis C is 2.5%, equivalent to 80 million infected people [1]. Although the incidence of hepatitis C virus (HCV) infection has decreased in some regions, such as Eastern Europe, Southern Africa and Australasia [2], mortality from HCV infection is expected to continue to increase over the next 20 years [3]. HCV infection is usually characterized by chronic infection that can progress for decades, leading to fibrosis, cirrhosis or liver cancer [4].

As a mechanism to fight viral infections, the immune response is able to produce factors that prevent viral replication and that destroy cells that serve as reservoirs. A characteristic of initial HCV infection is the production of interferon through the activation of Toll-like receptor 3 (TLR3) and retinoic acid-inducible gene-I (RIG-1), receptors that recognize the genetic material of the virus and initiate the production of interferons [5].

During the process of liver damage, when TLR3 activation occurs, Kupffer cells (KCs) induce the production of cytokines, such as IL-12, IL-18 and TNF-α, and the activation of natural killer (NK) cells. The interaction of receptors and cytokines stimulates NK cells to produce IFN-γ. The synergistic effect of IFN-γ and TNF-α produced by NK cells and KCs induces the death of hepatocytes [6]. Regarding HCV infection, studies of the primary cultures of infected human hepatocytes showed that when TLR3 is stimulated, there is mainly an antiviral response with high IFN-λ (IFN-III) production [7].

IFN-λ has antiviral activity in vitro [8,9] and in vivo [10,11]. Some authors have demonstrated this ability in some viruses, including HCV and hepatitis B viruses [9], the herpes simplex virus type 2 [10] and the human immunodeficiency virus 1 [12]. IFN-λ can exert its antiviral activity in HCV infection by suppressing miR-122 expression [13,14]. The production of IFN-α/β and IFN-λ induces an antiviral state in liver parenchyma cells, but for certain reasons, this is not enough to eliminate HCV infection. After 4–8 weeks, the viral load begins to decrease, and HCV-specific T cells begin to be recruited to the liver. At this time, the exacerbated production of IFN can lead to liver damage due to constant inflammation, increasing ALT levels and triggering the process of liver fibrosis [15].

In this sense, the present study evaluated *TLR3* and *IFNL3* gene expression levels in the livers of patients with chronic HCV infection, their correlation with the biochemical markers of liver damage (ALT, AST), bile duct obstruction (GGT), liver cancer (AFP), viral factors (viral load) and their association with the necroinflammatory activity profile in the liver as well as the degree of liver fibrosis.

## 2. Materials and Methods

### 2.1. Sampling and Study Design

This was a cross-sectional and analytical study. A total of 23 liver biopsy samples were obtained from the liver disease outpatient clinic of João de Barros Barreto University Hospital (HUJBB, for its acronym in Portuguese) and the Hospital of the Holy House of Mercy Foundation of Pará (FSCMPA, for its acronym in Portuguese), selecting consecutive cases of patients with chronic HCV for the period from August 2014 to January 2017.

The adopted inclusion criteria were individuals aged 18 years or older, of both sexes, with HCV RNA positivity for over 6 months and with the persistence of elevated or normal alanine aminotransferase levels. Individuals who did not meet the requirements stipulated above were excluded from the study, as were patients coinfected with HBV, HDV and/or HIV, according to their medical records, as well as patients who were using HCV-specific antiviral therapy.

The patients with chronic HCV were between 35 and 64 years of age, with a mean age of 52 years. Of these, 47.8% were female and 52.2% were male. Seventeen were infected with genotype 1, 7 were infected with subgenotype 1a, and 6 were infected with subgenotype 1b; in 4 patients, the subgenotype was undetermined. Only 2 patients were infected with genotype 3, whereas the viral genotype was not determined in the other patients (*n* = 4).

The control group (*n* = 8) that was used for the analysis of liver biopsies consisted of individuals who underwent conventional cholecystectomy without necroinflammatory liver changes at the surgery department of HUJBB.

### 2.2. Ethical Aspects

The present study was submitted to the Research Ethics Committee of the FSCMPA Hospital (No. 772,782), to HUJBB (No. 962,537) and to the Institute of Health Sciences of the Federal University of Pará (No. 684,432), according to resolution No. 466/2012 of the National Health Council/Ministry of Health. The patients selected for the study were informed about the study objectives, and those who agreed to participate signed an informed consent form.

### 2.3. Samples

Liver biopsies were obtained from patients with prothrombin activity greater than or equal to 70% and with a platelet count above 100,000 cells/mm^3^. The ultrasound-guided biopsies were performed by a medical professional using a Trucut needle. The liver biopsy samples were separated into 2 parts: 1 that was sent for genetic analysis at the Laboratory of Virology of the Institute of Biological Sciences (ICB) at the Federal University of Pará (UFPA), and 1 that was examined by the Department of Pathological Anatomy of UFPA, where the samples were subjected to hematoxylin and eosin (H&E), chromotrope aniline blue (CAB), Gomori’s reticulin and Shikata’s orcein staining, followed by histopathological diagnosis based on the classifications of the Brazilian Society of Hepatology and the French METAVIR classifications.

### 2.4. Virological and Biochemical Parameters of Liver Function

Data on alanine aminotransferase (ALT), aspartate aminotransferase (AST), gamma glutamyl transpeptidase (GGT) and alpha-fetoprotein (AFP) levels and the viral load were obtained from patient records.

### 2.5. RNA Extraction

The liver biopsy samples were stored in TRIzol (Thermo Fisher Scientific, Waltham, MA, USA) at −80 °C until use. Total RNA extraction was performed using the protocol recommended by the manufacturer.

The purity of each RNA sample was determined by the ratio between the absorbance measured at 260 nm and 280 nm using a NanoDrop 2000c spectrophotometer (Thermo Fisher Scientific, Waltham, MA, USA). A good extraction was defined as one in which the absorbance ratio was between 1.6 and 1.8. To observe the purity and integrity of RNA, the samples were loaded into a 1% agarose gel in 1× TAE buffer and subjected to 80 V electrophoresis for 90 min. The gel was then stained with ethidium bromide (0.5 μg/mL). The observed bands corresponded to the 28S, 18S and 5S rRNA subunits. Bands 28S and 18S appeared in the gel at a ratio of 2:1. The 5S band appeared as a weak band, indicating a low level of RNA degradation.

The RNA was quantified by spectrophotometry in a NanoDrop 2000c spectrophotometer. The concentration was adjusted to 30 ng/μL, and the RNA was stored at −70 °C until reverse transcription.

### 2.6. Reverse Transcription (cDNA)

Total RNA was transcribed into complementary DNA (cDNA) using a High-Capacity cDNA Reverse Transcription kit (without an inhibitor) according to the protocol provided by the manufacturer (Thermo Fisher, Carlsbad, CA, USA). The reaction was prepared in a final volume of 20.0 μL containing 4.2 μL of H_2_O, 2.0 μL of buffer, 2.0 μL of random primers, 0.8 μL of dNTP Mix (100 mM), 1.0 μL of reverse transcriptase (RT) enzyme and 30 μL of RNA (30 ng/μL). The solution was then placed into a thermocycler with the following program: 25 °C for 10 min, 37 °C for 120 min and 85 °C for 5 min.

### 2.7. TLR3 and IFNL3 Expressions Determined by Real-Time PCR (qPCR)

Real-time PCR was performed in 96-well plates using TaqMan^TM^ reagents (Applied Biosystems, Waltham, MA, USA) and a Step One Plus real-time PCR system (Applied Biosystems, Foster City, CA, USA). The probes for the target genes and endogenous control were obtained from ThermoFisher (Carlsbad, CA, USA). The TaqMan^®^ Gene Expression Hs01551078_m1 assay, the Hs04193049_gH assay and the Hs02786624_g1 assay were used for *TLR3*, *IFNL3* and *GAPDH* (internal control), respectively.

Each reaction consisted of 15 μL of TaqMan 2× Universal PCR Master Mix, 1.5 μL of TaqMan 20× Gene Expression Assay solution, 3 μL of cDNA and 10.5 μL of RNase-free water. The thermocycler was programmed for 1 cycle of 2 min at 50 °C, followed by 10 min at 95 °C and 40 cycles at 95 °C for 15 s and at 60 °C for 1 min. All reactions were performed in duplicate. The relative quantification of the target genes was performed using the comparative CT method (ΔΔCT).

### 2.8. Statistical Analysis

The distribution of the gene expression and biochemical marker data was evaluated using the Shapiro–Wilk test; whenever a normal distribution was confirmed, the data were compared using ANOVA. Correlation analysis was used to evaluate the association between the viral load and the gene expression (Pearson linear correlation). The comparison of gene expression levels between groups was performed using the non-parametric Mann–Whitney test.

Heatmaps were constructed using the heatmap.2 function in the R program (Project for Statistical Computing, version 3.4.1).

Biomarker networks were designed to evaluate the correlations between the biomarkers that were analyzed; Cytoscape software was used for this purpose [16]. The networks were constructed using edges that represented the correlation scores, which were categorized as strongly positive (r ≥ 0.68; continuous bold line), moderately positive (0.36 ≥ r ≤ 0.67; continuous thin line) or negative (−0.37 ≥ r; dashed line).

A value of *p* < 0.05 was considered statistically significant. Statistical analyses were performed using a GraphPad Prism 5.

## 3. Results

### Expressions of TLR3 and IFNL3 Relative to HCV Genotype and Viral Load

**Figure 1:**
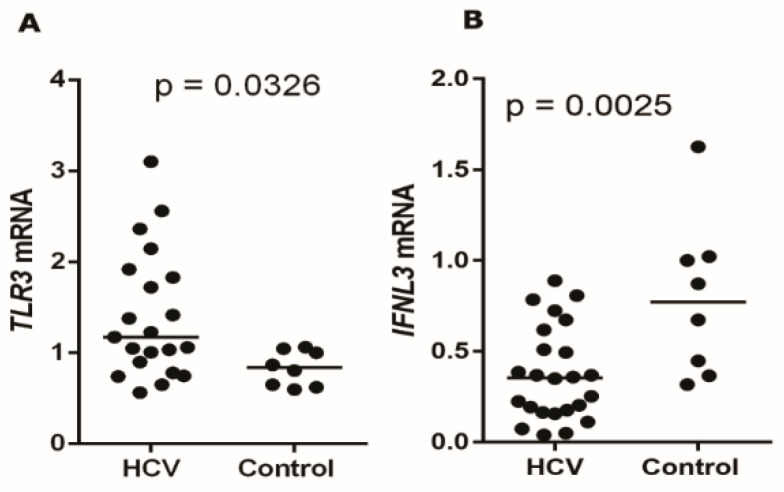
Median intrahepatic gene expressions of *TLR3* (**A**) and *IFNL3* (**B**) for groups of patients chronically infected by HCV and those of healthy controls. Median *TLR3*: HCV = 1.08 and control = 0.86; median *IFNL3*: HCV = 0.43 and control = 0.86. Mann–Whitney Test.

The gene expression analysis showed that compared to the controls, patients with chronic HCV infection had a higher intrahepatic expression of *TLR3* (Figure 1A; *p* = 0.0326; medians: HCV = 1.08 and control = 0.86) and a lower expression of *IFNL3* (Figure 1B; *p* = 0.0025; medians: HCV = 0.43 and control = 0.86).

**Figure 2:**
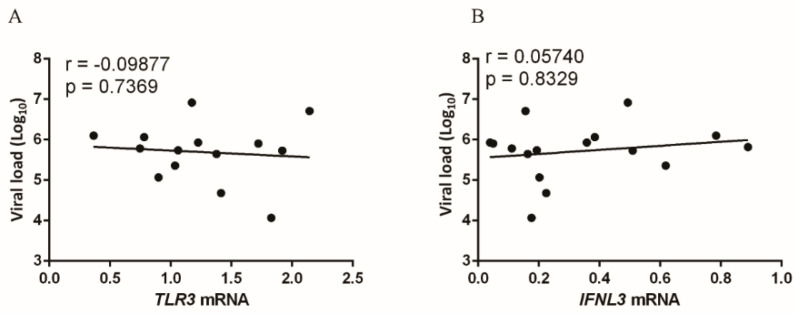
Plasma viral load of patients and its correlation with the intrahepatic expression of *TLR3* (**A**) and *IFNL3* (**B**). Pearson linear correlation.

To understand how the serum HCV viral load may be correlated with the expression of the analyzed markers, a correlation analysis of the serum viral load of the patients with the intrahepatic expression of *TLR3* (Figure 2A) and *IFNL3* (Figure 2B) was performed. The r values were close to zero, with a nonsignificant *p* value, indicating that there was no correlation.

## 4. Necroinflammatory Activity and Staging of Liver Tissue Fibrosis Relative to *TLR3* and *IFNL3* Expressions, Viral Enzymes and HCV Genotype

**Figure 3:**
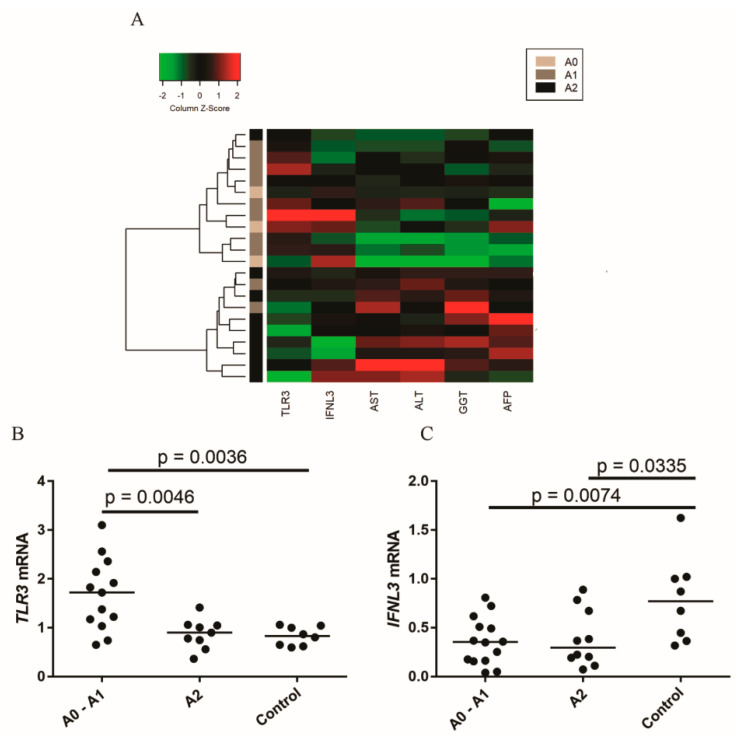
(**A**) Heatmap of the intrahepatic gene expressions of *TLR3* and *IFNL3* and the levels of biochemical markers (AST, ALT, GGT and AFP) relative to the liver necroinflammatory activity in patients. Comparison of the expression levels of (**B**) *TLR3* and (**C**) *IFNL3* among patients with different degrees of necroinflammatory activity and the control group. Mean *TLR3*: A0-A1 = 1.8, A2 = 0.84 and control = 0.81; mean *IFNL3*: A0-A1 = 0.47, A2 = 0.44 and control = 0.85. ANOVA test.

A heatmap was constructed for the joint analysis of necroinflammatory activity scores, intrahepatic *TLR3* and *IFNL3* expression levels, viral enzymes and HCV genotypes (Figure 3A). The heatmap showed the formation of two clusters. The first cluster comprised patients with METAVIR A0 and A1 scores who had a high intrahepatic expression of *TLR3* and *IFNL3* and low levels of liver transaminases. The second cluster comprised patients with METAVIR A2 scores who had higher transaminase levels and a lower intrahepatic expression of *TLR3* and *IFNL3*.

To reinforce the findings that were found in the heatmap, the intrahepatic expressions of *TLR3* (Figure 3B) and *IFNL3* were analyzed based on the necroinflammatory activity score (Figure 3C). Regarding *TLR3* expression, the control group exhibited a basal expression of this receptor; however, in the A0–A1 group, the expression was significantly higher than that in the other groups (means: A0–A1 = 1.80, A2 = 0.84 and control = 0.81). For the A2 group, *TLR3* expression was re-established, remaining similar to that in the control group. Figure 3C shows that patients with A0–A1 and A2 scores had lower levels of *IFNL3* expression than did the control group (means: A0–A1 = 0.47, A2 = 0.44 and control = 0.85).

**Figure 4:**
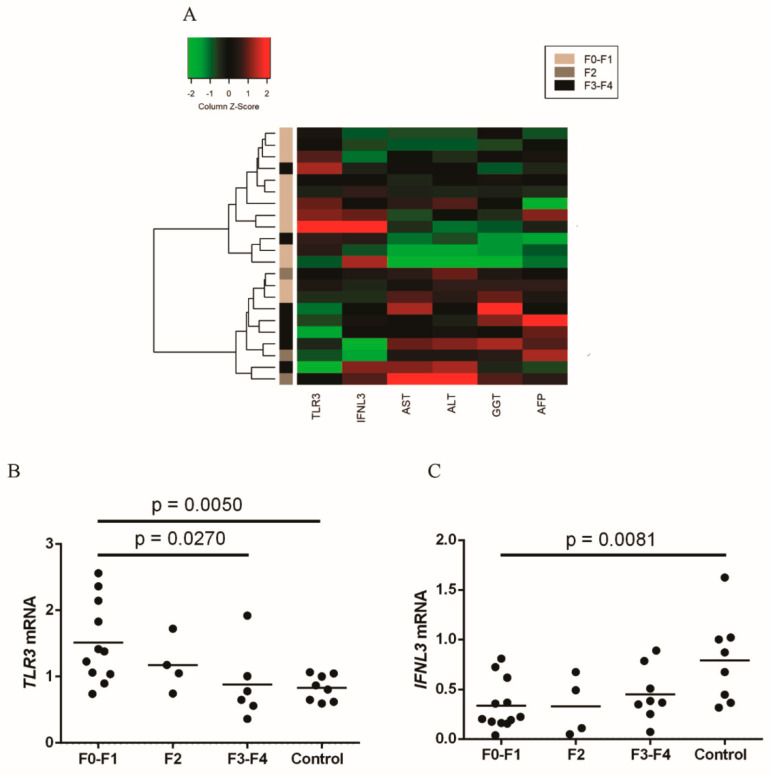
(**A**) Heatmap of the intrahepatic gene expressions of *TLR3* and *IFNL3* and levels of biochemical markers (AST, ALT, GGT and AFP) relative to the liver fibrosis of patients. Comparison of the expression levels of (**B**) *TLR3* and (**C**) *IFNL3* among patients with different fibrosis scores and the control group. Mean *TLR3*: F0-F1 = 1.81, F2 = 1.26, F3-F4 = 0.96 and control = 0.84; mean *IFNL3*: F0-F1 = 0.42, F2 = 0.41; F3-F4 = 0.47 and control = 0.85. ANOVA test.

The heatmap for fibrosis scores and for the other investigated variables showed a result similar to that observed in the evaluation of necroinflammatory activity, with two clusters: patients with METAVIR F0–F1 scores had higher intrahepatic expressions of *TLR3* and *IFNL3* and low levels of liver transaminases, whereas patients with higher fibrosis scores had higher transaminase levels and lower intrahepatic expressions of *TLR3* and *IFNL3* (mean *TLR3*: F0–F1 = 1.81, F2 = 1.26, F3–F4 = 0.96 and control = 0.84; mean *IFNL3*: F0–F1 = 0.42, F2 = 0.41; F3–F4 = 0.47 and control = 0.85). The intrahepatic expression of *TLR3* was higher in patients in the F0–F1 group (Figure 4B), and the expression of *IFNL3* was higher in the control group (Figure 4C).

### Interaction Networks between the Analyzed Markers

A network of interactions between the study variables was built through Pearson′s correlations, including only patients who possessed all of the data on the viral load; AST, GGT, ALT and AFP levels; and *TLR3* and *IFNL3* gene expressions.

**Figure 5:**
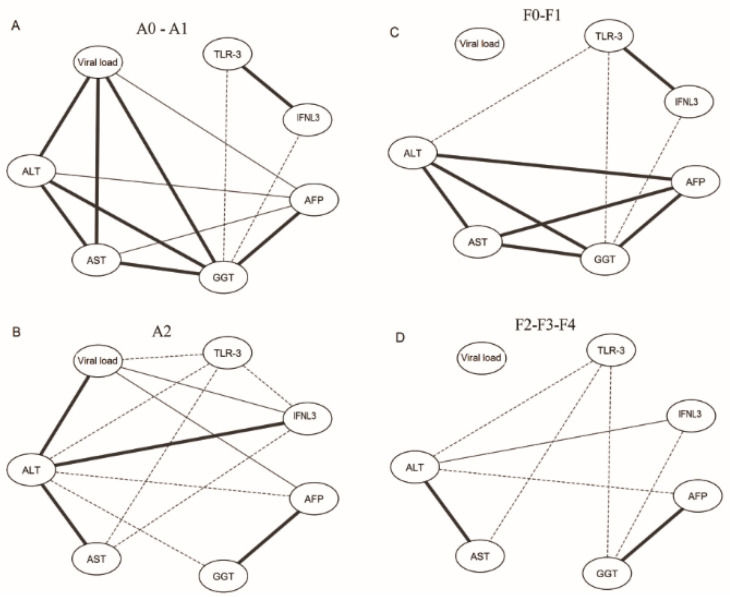
Interaction networks for the biomarkers analyzed among patients with degrees of necroinflammatory activity (**A**) A0-A1 and (**B**) A2; fibrosis scores (**C**) F0-F1 and (**D**) F2-F3-F4. Correlation values were categorized and are represented by the connecting lines (edges). The r values were used to categorize the correlations into strongly positive (r ≥ 0.68; solid bold line), moderately positive (0.36 ≥ r ≤ 0.67; continuous thin line) and negative (−0.37 ≥ r; dashed line). Pearson correlation.

We observed an increase in liver enzymes in the early stages of inflammation (A0–A1) and liver fibrosis (F0–F1). For patients with scores of A0–A1, the viral load showed a strong positive correlation (solid bold line) with the serum levels of the transaminases ALT, AST and GGT and a moderate positive correlation with AFP (continuous thin line). The serum HCV viral load was not correlated with the intrahepatic expressions of *TLR3* and *IFNL3* (dashed line). There was a strong positive correlation between the expression levels of *TLR3* and *IFNL3* and a negative correlation between the expression levels of *TLR3* and *IFNL3* and serum GGT levels (Figure 5A).

In patients with scores of A2, the serum viral load had a positive correlation with the serum levels of ALT and AST, in addition to a negative correlation between the viral load and intrahepatic *TLR3* expression and a negative correlation between *TLR3* and *IFNL3*. However, a moderate positive correlation was found between *IFNL3* and the viral load. The correlations between GGT and AFP and between AFP and the viral load were still present (Figure 5B).

Patients with scores of F2-F3–F4 were grouped into the same analysis due to the small sample size, and these patients had a similar expression profile and marker levels, which were clustered in the heatmap. Regarding the degree of fibrosis, the network graph showed that the serum viral load was not correlated with the fibrosis score (F0–F1 and F2-F3–F4). However, in the early stages of fibrosis (F0–F1), *TLR3* expression had a strong positive correlation with *IFNL3* expression, and there was also a negative correlation between *IFNL3* and transaminases. The liver transaminases (AST, ALT, GGT and AFP) were strongly correlated with each other (Figure 5C). In patients with scores of F2-F3–F4, *TLR3* and *IFNL3* expressions were no longer correlated. However, the enzymes ALT, AST, GGT and AFP showed strong correlations (Figure 5D).

## 5. Discussion

### 5.1. Expression of TLR3 and IFNL3 Relative to HCV Genotype and Viral Load

There have been several liver tissue studies that associated TLR3 with intense inflammation and liver damage [6,17]. In the liver, TLR3 activation leads to higher IFN-λ production [7], which can inhibit HCV replication [18]. Due to the substantial importance of the TLR3 signaling pathway in liver tissue, we evaluated the intrahepatic expression of *TLR3* and its activation product, *IFNL3*.

In this study, the expression levels of *TLR3* and *IFNL3* were higher and lower, respectively, in patients with chronic HCV than in the healthy control group, corroborating the results of previous studies [19,20,21]. These results are associated with the physiology of HCV infection; i.e., TLR3 detects the intermediate form of dsRNA (secondary RNA segment) during HCV replication [18]. The activation of TLR3 in liver cells induces the activation of IRF-3 and the robust synthesis of ISGs [18] and IFN-λ [22], which inhibits HCV replication. However, as an avoidance mechanism, HCV is able to inhibit TLR3 signaling by suppressing TRIF expression. This inhibition of TLR3 may be an important factor for HCV to establish a persistent infection [18]. Thus, although we found a high *TLR3* expression in the liver tissue of patients with chronic HCV, we observed a lower *IFNL3* expression, probably due to viral evasion mechanisms.

There is a positive correlation between the proportion of infected hepatocytes, the serum and the intrahepatic viral load in HCV-infected patients [23,24]. Thus, the correlation of the serum viral load with the intrahepatic expressions of *TLR3* and *IFNL3* was analyzed to visualize how these markers could interact in liver tissue. However, no significant results were observed, a result that does not exclude the possibility that HCV evades the immune response and significantly controls the expression of these markers.

Other studies have also described the lack of a correlation between the expression of *TLR3* in the peripheral blood, the serum viral load and the degree of fibrosis in patients infected with HCV [25,26,27], and no correlation has been observed between ISG expression in the liver and the number of infected hepatocytes or the serum viral load [23].

A previous study reported that although the serum HCV viral load is not correlated with the intrahepatic *TLR3* expression, the intrahepatic viral load is positively correlated with this gene [24]. Thus, they suggested that the serum viral load does not correctly reflect the intrahepatic viral load because the concentration of viral particles in the serum may be altered by several factors, such as the peripheral immune response.

### 5.2. Necroinflammatory Activity and Staging of Liver Tissue Fibrosis Relative to TLR3 and IFNL3 Expressions, Viral Enzymes and HCV Genotypes

Liver necroinflammatory activity scores range from 0 to 3 and indicate the rate of progression of the liver fibrosis process, as they evaluate the presence of inflammatory cells between the liver lobules. In this study, the results showed that patients with an absent or mild necroinflammatory process (METAVIR A0 and A1 score) had high intrahepatic *TLR3* and *IFNL3* expressions as well as low liver transaminase levels. In addition, there was a strong positive correlation between *TLR3* and *IFNL3*, indicating that in this more stable stage of chronification, they are important in the inflammation process and in the maintenance of the antiviral state. TLR3 seems to act as an important sensor for HCV RNA that is present at the site of HCV infection, and type III IFN is one of the products resulting from the TLR3 signal in response to exogenous dsRNA [28,29]. These data indicate that high levels of these markers are an initial attempt by the body to fight infection without causing severe liver damage.

The network graphs show that in a stable process of chronification of an HCV infection, during which the liver parenchyma has little inflammatory cell infiltrate (A0–A1), the virus (represented by the serum viral load) is the main causative agent of the inflammatory process, moderately increasing the serum levels of liver transaminases (ALS, AST and GGT). However, the serum HCV viral load is not correlated with the increase in intrahepatic *TLR3* and *IFNL3* expressions. The relationships between the viral load, degree of inflammation and fibrosis score seem to vary between different studies, which have shown no association [30] or a correlation with more advanced stages of the disease [31]. These results suggest that the available data on viral load indicate that this marker does not predict the severity of chronic hepatitis C.

There was a negative correlation between *TLR3* and *IFNL3* and the serum levels of GGT, suggesting that these two markers can efficiently suppress virus replication and influence the levels of one of the main characteristics of HCV infection: cholestasis (blockage of the bile ducts). Approximately 5% of chronic HCV patients will develop severe cholestasis, and this condition is associated with a high viral load and the presence of an intrahepatic Th2 cytokine profile [32]. However, as chronic intrahepatic disease is multifactorial, other co-factors, such as obesity and alcohol, can modulate the risk of fibrosis progression, and this association cannot be sustained [33].

The importance of *TLR3* expression levels in the progression of infection was also demonstrated through an analysis of peripheral blood. Patients who had spontaneously cleared HCV had higher levels of receptor expression [34], indicating that in an initial inflammation process, the HCV-mediated immune response is extremely important for the resolution of infection.

Our results show that patients with a more severe necroinflammatory process (METAVIR A2 score), which is usually characteristic of a faster progression of liver fibrosis, have higher transaminase levels and lower intrahepatic *TLR3* and *IFNL3* expressions. However, as liver fibrosis progresses to the F2-F3–F4 phases, the loss of the correlation between the expressions of *TLR3* and *IFNL3* with a predominance of the GGT enzyme is evident, attributed to the presence of lesions in the bile ducts [32] and positively correlated with the METAVIR score [35] in patients with HCV.

We observed that the virus (represented by the serum viral load) is still primarily responsible for the inflammatory process leading to increased serum levels of ALT and AST. However, there is a loss in the ability of the immune system to control this infectious process, probably due to exhaustion and/or viral evasion mechanisms and advanced liver damage, as evidenced by a negative correlation between the viral load and intrahepatic *TLR3* expression. There was also a negative correlation between *TLR3* and *IFNL3*.

The decrease in the activation of the studied genes is due to the inhibition of TLR3 signaling via several viral evasion mechanisms used by HCV [22,36,37,38,39,40]. Some data support these findings because in PBMCs, the production of IL-6 and TNF-α via TLR3 stimulation is impaired in patients with chronic HCV [27].

In conclusion, the results suggest that in the early stages of the development of hepatic fibrosis, TLR3 and IFN-λ3 play important roles in the antiviral response and in the modulation of the tolerogenic liver environment because there is a decrease in the intrahepatic expressions of *TLR3* and *IFNL3* in the advanced stages of fibrosis due to the severe liver damage caused by HCV. Finally, considering the small sample size of the present study, we suggest conducting further studies with larger cohorts and from different ethnic groups in order to confirm our findings.

## Data Availability

The data presented in this study are available on request from the corresponding author. The data are not publicly available due to restrictions of privacy and ethical.
